# Gold Nanoparticles (AuNPs)—Toxicity, Safety and Green Synthesis: A Critical Review

**DOI:** 10.3390/ijms25074057

**Published:** 2024-04-05

**Authors:** Łukasz Niżnik, Maciej Noga, Damian Kobylarz, Adrian Frydrych, Alicja Krośniak, Lucyna Kapka-Skrzypczak, Kamil Jurowski

**Affiliations:** 1 Department of Regulatory and Forensic Toxicology, Institute of Medical Expertise, Łódź, ul. Aleksandrowska 67/93, 91-205 Łódź, Polandtoksykologia@ur.edu.pl (K.J.); 2 Laboratory of Innovative Toxicological Research and Analyses, Institute of Medical Studies, Medical College, Rzeszów University, Al. mjr. W. Kopisto 2a, 35-959 Rzeszów, Poland; 3 Department of Molecular Biology and Translational Research, Institute of Rural Health, 20-090 Lublin, Poland; 4 World Institute for Family Health, Calisia University, 62-800 Kalisz, Poland

**Keywords:** nanotechnology, toxicology, gold nanoparticles, green synthesis, nanoparticle toxicity

## Abstract

In recent years, the extensive exploration of Gold Nanoparticles (AuNPs) has captivated the scientific community due to their versatile applications across various industries. With sizes typically ranging from 1 to 100 nm, AuNPs have emerged as promising entities for innovative technologies. This article comprehensively reviews recent advancements in AuNPs research, encompassing synthesis methodologies, diverse applications, and crucial insights into their toxicological profiles. Synthesis techniques for AuNPs span physical, chemical, and biological routes, focusing on eco-friendly “green synthesis” approaches. A critical examination of physical and chemical methods reveals their limitations, including high costs and the potential toxicity associated with using chemicals. Moreover, this article investigates the biosafety implications of AuNPs, shedding light on their potential toxic effects on cellular, tissue, and organ levels. By synthesizing key findings, this review underscores the pressing need for a thorough understanding of AuNPs toxicities, providing essential insights for safety assessment and advancing green toxicology principles.

## 1. Introduction

The increasing incorporation of AuNPs in diverse fields, such as biomedicine, where they are used for drug delivery systems, diagnostics, and therapeutic agents, electronics, in which they contribute to improving sensors, conductivity, and efficiency of electronic devices, and environmental applications, including water purification and pollutant detection, represents a pivotal shift towards nanotechnology-enhanced solutions [1,2,3]. This widespread adoption is mainly due to AuNPs’ unique physico-chemical properties, which offer unprecedented opportunities for innovation across these sectors. However, the properties that make AuNPs valuable also require thorough examination of their potential health and environmental impacts [4,5].

The size of AuNPs is critical in determining their biological behaviour and toxicity [4,6,7,8]. Nanoparticles, by definition, possess at least one dimension less than 100 nm. This small size facilitates their penetration through biological barriers via self-assembly processes, allowing them to reach sites inaccessible to the human body and the environment [9]. However, this also raises concerns about their ability to induce cellular damage, leading to cytotoxicity, genotoxicity, and inflammatory responses. Research has shown that smaller AuNPs can more easily enter cells and accumulate in various organs, including the liver, spleen, and brain, posing potential health risks [10,11,12].

Another essential aspect of AuNPs is their shape, which influences their interaction with biological systems [13,14]. Various shapes, such as rods, spheres, and cubes, exhibit different surface areas and aspect ratios, affecting their cellular uptake and distribution within organisms [15]. For example, rod-shaped nanoparticles have been shown to exhibit biodistribution patterns different from those of spherical nanoparticles, which can impact their toxicity and efficacy in biomedical applications [16].

Another aspect of the review of AuNPs safety is the safety assessment, a critical component of their development and application. This ensures that these nanomaterials can be used safely in various fields without posing undue risks to human health when implementing nanoparticles as a potential treatment in clinical trials [17,18] or in the environment. This process is comprehensive and multi-faceted, incorporating a variety of in vitro (test tube or cell culture) [19,20] and in vivo (living organism) [21] studies designed to thoroughly investigate the potential health hazards associated with exposure to AuNPs. Through these studies, researchers aim to understand the immediate toxicological effects and long-term implications of exposure to these nanoparticles.

However, green toxicology, an emerging paradigm, emphasizes the design and synthesis of AuNPs that are inherently safer by design [22,23,24]. This approach advocates for incorporating toxicological considerations into the early stages of nanoparticle development, aiming to minimize adverse health and environmental impacts without compromizing the functional benefits of AuNPs. The principles of green chemistry and green engineering are crucial in guiding the synthesis of AuNPs that are safe, efficient, and environmentally benign [24,25].

The problem of AuNPs toxicity is a complex and evolving field that requires a holistic approach to safety assessment. By integrating advanced analytical methodologies, in-depth toxicological studies, and the principles of green toxicology, researchers and policymakers can ensure the responsible development and use of AuNPs. The collective effort to understand and mitigate the toxicological risks associated with AuNPs will pave the way for their sustainable application in various industries, ultimately contributing to public health safety and environmental preservation. Therefore, the overarching idea of this review is to synthesize and critically evaluate the existing body of research on the toxicological profiles of AuNPs, their safety assessment methodologies, and the integration of green toxicology principles into their development and application. This comprehensive review highlights the intricate balance between the use of AuNPs in various sectors and the potential risks their use poses to human health and the environment.

## 2. Materials and Methods

### 2.1. Search for Publications for Publications with Data on Toxicological Aspects, Safety Assessment, and Green Toxicology of Gold Nanoparticles

A multi-faceted data collection methodology was used to comprehensively investigate the toxicological aspects, safety assessment, and green toxicology of AuNPs. The research used a variety of scientific databases, including Web of Science and Scopus [26], Google Scholar [27], and PubMed [28]. These platforms were instrumental in accessing a large repository of scientific literature that included peer-reviewed articles, conference proceedings, and review articles across disciplines such as toxicology, nanotechnology, environmental sciences, and medicine. The use of these databases ensured a broad and diversified coverage of existing research on AuNPs, facilitating a thorough review of the literature. In addition to traditional scholarly sources, the investigation incorporated ‘grey’ literature [29], which included reports, theses and internet discussion forums. This inclusion was based on the understanding that this ‘grey literature’ could offer valuable insights and preliminary findings and articulate community concerns that could not yet be captured in peer-reviewed publications. This comprehensive approach recognized the importance of integrating diverse perspectives and data sources to construct a holistic view of the toxicological profile of AuNPs. We employed a strategic combination of advanced digital tools to execute our study’s methodology. Covidence [29], Consensus [30], and SysRev [31] streamline the process of identifying, selecting, and reviewing the most pertinent literature on the toxicological aspects, safety assessment, and green toxicology of AuNPs. Integrating these tools into our research process was crucial, allowing a highly organized and efficient review of an extensive body of literature. This approach significantly improved our ability to manage the complexities of synthesizing large amounts of data from various sources.

### 2.2. Keywords and Selection of Scientific Data

In the comprehensive methodology used for our study on AuNPs, a meticulous process was implemented to identify and select scientific data, underpinned by the strategic use of keywords and a structured review process. The initial phase of data collection involved the formulation of a broad yet targeted search strategy, employing a combination of keywords that included ‘AuNPs’, ‘gold nanoparticles’, ‘green synthesis’, ‘GS’ (an abbreviation commonly used in the context of green synthesis of metallic nanoparticles), ‘metallic nanoparticles’, ‘natural metallic nanoparticles’, ‘toxicity’, ‘green toxicology’, ‘toxicological aspects’, ‘safety’, and ‘safety assessment’. This extensive list of terms was carefully chosen to encapsulate the multifaceted nature of our research focus, ensuring the inclusion of literature that spans the spectrum from synthesis methods to the implications of AuNPs in toxicology and safety.

The process of selecting appropriate studies was meticulously divided into two distinct stages, designed to rigorously filter and identify the most relevant research contributions for our review. The first stage involved a brief overview of the titles and abstracts of the findings. This preliminary selection served as an initial filter to discern the relevance of studies based solely on their titles and abstracts, allowing the exclusion of unrelated or tangential papers from the pool of potential sources. To enhance the objectivity and comprehensiveness of this screening phase, each author independently reviewed the titles and abstracts at different times, ensuring that the selection process benefitted from diverse perspectives and reducing the likelihood of oversight.

### 2.3. Classification of Results

In our study methodology, we refined the classification of results to focus exclusively on sources directly relevant to the toxicological profiles of AuNPs. This strategic narrowing was essential to understand better our research’s core objectives: to dissect the complex interactions and potential hazards of AuNPs in biological systems and the environment. To this end, we defined specific inclusion criteria for our literature review, targeting studies that delved into the presence, function, and adverse impacts of AuNPs in several key dimensions.

The inclusion criteria included studies on the in vitro and in vivo toxicology of AuNPs, which highlight their biological interactions and potential toxic effects. We also focused on research assessing the toxicity of AuNPs against immune cells and normal human cell lines to gauge their safety profile for potential biomedical applications. Additionally, our review prioritized studies reporting adverse effects of AuNPs, organ-specific toxicity, and the underlying mechanisms driving such toxicological outcomes, including both oxidative and non-oxidative stress pathways.

This targeted approach facilitated the accumulation of a comprehensive dataset, shedding light on the multifaceted nature of AuNPs toxicity. By concentrating on these areas, our methodology ensured a systematic and focused literature review, laying a solid foundation for a nuanced understanding of the safety and environmental implications of AuNPs use. This process was instrumental in guiding our analysis towards findings critical for evaluating the risk-benefit ratio of AuNPs, thereby contributing to the development of safer nanotechnologies.

### 2.4. Presentation of the Results

In our study of AuNPs, we organized the findings into three succinctly defined sections to enhance clarity and readability, focussing on distinct but interrelated aspects of AuNP research:

1. Toxicological Aspects of AuNPs: This section thoroughly examines AuNPs’ interactions with biological systems, covering their toxicological in vitro and in vivo impacts. It includes insights into cellular uptake, biodistribution, and toxicity mechanisms, such as oxidative and non-oxidative stress pathways. The analysis extends to the effects of AuNPs on immune cells, normal human cell lines, and organ-specific toxicity, offering a comprehensive view of their biological interactions and potential health risks.
2. Safety Assessment of Gold Nanoparticles in Cosmetic Products: We assess the implications of using AuNPs in cosmetics and their safety. This segment also delves into regulatory frameworks and safety standards governing the use of nanoparticles in personal care products in the EU.
3. Green Toxicology of Gold Nanoparticles: Dedicated to the environmental aspect, this section explores the integration of green chemistry principles into the synthesis and lifecycle of AuNPs. It focuses on reducing the environmental impact through sustainable practices, including biodegradability and recyclability, highlighting the importance of environmental stewardship in developing and applying nanotechnologies.

Each section addresses specific aspects of AuNPs research, from their biological and environmental interactions to their safe use in consumer products. Thus, this approach facilitates a holistic understanding of AuNPs and promotes informed discussions on their sustainable and responsible use.

### 2.5. Uncertainties or Limitations of Review

During our comprehensive review of AuNPs, we encountered several uncertainties and limitations that have implications for the interpretation and generalisation of our findings. The inherent heterogeneity of the included studies, which span various methodologies, nanoparticle characteristics, and biological models, complicates the synthesis of results and constrains the ability to formulate overarching conclusions about toxicology and safety. This variability underscores the challenge of comparing studies directly, due to differences in experimental conditions, exposure levels, and endpoints evaluated.

A notable limitation of the existing literature is the predominance of short-term and in vitro evaluations, which, while providing essential information on immediate toxicological effects and potential mechanisms of action, fall short of capturing the long-term impacts and in vivo behaviour of AuNPs. The lack of comprehensive long-term exposure and in vivo studies marks a significant gap in our understanding of these nanoparticles’ chronic effects and biodistribution upon environmental or biological exposure.

Furthermore, the review highlights a lack of standardisation in the synthesis and characterisation of AuNPs in all studies. Variations in nanoparticle production and characterisation techniques lead to a wide range of physico-chemical properties, influencing their biological interactions and complicating the reproducibility and comparability of research findings.

Regarding green toxicology, our review reveals an insufficiency of studies that systematically address the environmental impacts of AuNPs throughout their lifecycle. Research on the degradation, accumulation, and persistence of AuNPs in environmental matrices is critical for future investigations in order to ensure nanoparticle technologies’ environmental safety and sustainability.

The evolving regulatory landscape and variability in nanoparticle safety assessment guidelines also present challenges. The absence of universally accepted standards to evaluate AuNPs safety in different applications, including cosmetics, underscores the need for harmonized safety assessments and regulatory frameworks.

These limitations and uncertainties highlight the need for ongoing research efforts to standardize methodologies, promote long-term and in vivo studies, and expand the scope of green toxicology research. Collaboration among scientists, industry stakeholders, and regulatory bodies will be essential in developing comprehensive and universally accepted guidelines for the safe and sustainable application of AuNPs. By addressing these gaps, future work can build on the foundation laid by our review, advancing our understanding of the safety and environmental impact of AuNPs.

## 3. Toxicological Aspects of Gold Nanoparticles

### 3.1. In Vitro Toxicology Studies on AuNPs

The predominant focus of assessments in nanotoxicology is on those conducted in vitro, due to their simplicity and ease of execution. However, it is recognized that in vitro evaluations may not accurately anticipate in vivo toxicity [32]. Despite this limitation, in vitro studies provide fundamental information on uptake and toxicity mechanisms, as shown in Table 1, with key studies.

AuNPs have been implicated in inducing imbalances in oxidative status in in vitro settings [55,69]. The present investigation reveals that exposure of hepatocytes to AuNPs results in a time- and dose-dependent increase in reactive oxygen species (ROS) production, with the highest dose causing more extensive damage. These findings are corroborated by cell viability assays, indicating an increase in cell mortality. Consequently, AuNPs initiate initial oxidative damage, prompting cells to regulate ROS over-production, potentially attributable to enhanced antioxidant activity [64,70]. Similarly, cell viability assays involving human colorectal adenocarcinoma cells (HT29) exposed to AuNPs exhibit a notable decrease in viable cells without concurrent genotoxic effects [57]. HepG2 cells exposed to 10-nm AuNPs show comet tails similar to those induced by a positive control involving hydrogen peroxide, indicating the potential entry of 10 and 30-nm nanoparticles into the nucleus, validated by NPs within the nucleus [54]. Contrary to expectations, AuNPs treatments do not alter the concentration of inflammatory markers compared to control [54], which is consistent with findings from other authors [71,72,73,74]. The authors posit that the size of nanoparticles dictates their route of excretion. Moreover, it has been confirmed that AuNPs trigger disruptions in the oxidative equilibrium of cells, resulting in molecular impairments, such as genetic, protein, and lipid damage. Additionally, A549 cells exhibit an inflammatory reaction to 20 nm AuNPs. [37]. AuNPs display minimal long-term toxicity in MG63 cells [62]. Guglielmo et al. [39] assessed the toxicity of both uncoated and coated spherical AuNPs in BALB/c 3T3 cells, demonstrating that DNA damage arises from indirect oxidative stress. Various studies underscore the genotoxic potential of AuNPs, with evidence of DNA damage in airway epithelial cells, accompanied by elevated lipid peroxidation and cytotoxicity [44]. Predictive models involving the association of 12 various NPs with DNA indicate that AuNPs possess a high affinity for DNA, suggesting a more pronounced inhibitory property in DNA replication than other NPs [75]. The epithelial cells’ fine airways exhibit a notable increase in lipid peroxidation, DNA impairments, and cytotoxicity [44]. While studies extensively explore size- and time-dependent DNA damage caused by AuNPs, scant attention has been paid to dose-dependent DNA damage. Some investigations report DNA damage post-exposure to 8 nm AuNPs [76] and 20 nm AuNPs [77]. The damage originates from the strong attraction between AuNPs, thiol, and the amine group, resulting in interactions with biomolecules and intense radical creation. [78,79]. These nano-scaled particles, with immense surface areas, can instigate the production of reactive oxygen species (ROSs). These ROSs induce cellular damage, impacting proteins, DNA, membranes, and various organelles, such as the cytoplasm, mitochondria, and nucleus [80]. Numerous studies emphasize the toxicities of AuNPs induced by ROS production. Das et al. [63] observed mild hepatotoxic and nephrotoxic effects in MRC-5 cells exposed to AuNPs capped with trisodium citrate dihydrate (AuNPC) and bovine serum albumin (AuNPB). Investigations in human lung fibroblast MRC-5 revealed that AuNPs induced substantial lipid peroxidation and upregulated antioxidants and elicited the expression of stress response proteins and genes [64,70].

Khan et al. [66] found notable levels of lipid peroxidation in rat livers exposed to AuNPs. Mateo et al. [55] established the harmful effects of three differently sized AuNPs on human leukemia (HL-60) and hepatoma (HepG2) cell lines, showing decreased levels of glutathione (GSH) after 72 h of exposure alongside increased production of reactive oxygen species (ROSs). GSH-capped AuNPs exhibited higher ROS production than plain AuNPs in 3T3 cells [33]. Citrate-capped AuNPs demonstrated toxicity in HeLa and U937 cells, with the effects dependent on the cell culture medium [48,49], while sodium citrate residues influenced the cytotoxicity of AuNPs on human epithelial cells [81]. Martinez Paino et al. [53] observed in vitro toxicity and genotoxicity of sodium citrate- or Z-capped AuNPs dendrimers at low concentrations in HepG2 and PBMC cells. Oxidative stress resulted in DNA damage in Balb/3T3 cells treated with uncoated and hyaluronic acid-coated AuNPs, although their internalization and toxicity were reduced [39]. High concentrations of AuNPs was responsible for oxidative stress affecting cell viability and causing actin and tubulin deformations in C17.2 and PC12 cells [41]. Treatment of Balb/3T3 cells with citrate-stabilized AuNPs led to cytotoxicity through alteration of the actin cytoskeleton [40]. In comparison to AuNPs stabilized with gum Arabic and starch, citrate-coated AuNPs showed greater cytotoxicity due to their citrate-acidic properties [82]. Indications of autophagy and oxidative stress were observed in MRC-5 cells exposed to 20 nm AuNPs [64,70]. The type of cell death induced by AuNPs varies, with smaller sizes causing rapid necrosis (1.4 nm) or apoptosis (1.2 nm) [83]. Surface charge also influences cell death mechanisms, as evidenced by the involvement of apoptosis and necrosis in human keratinocyte cell line HaCaT treated with 1.5 nm AuNPs [46]. It is noteworthy that AuNPs can induce cell death through various mechanisms depending on cell type. Chueh et al. [65] noted reduced growth in Vero, MRC-5, and NIH/3T3 cells due to apoptosis and autophagy. Multiple studies support the notion that NPs interact with membrane lipids, leading to adverse effects on cells.

Leroueil et al. [84] stated that NH2-AuNPs caused physical damage to lipid membranes, as observed by atomic force microscopy. The association of different AuNPs with bovine serum albumin (BSA) showed that BSA preserved its characteristics, except for AuNRs, which resulted in a significant loss of secondary and tertiary structures of the protein [85]. AuNPs with a diameter of 10 nm increase the accumulation of estrogen within granulose cells of the ovary after an incubation period of 1–5 h [45]. Furthermore, these nanoparticles can enter and modify certain internal cellular organelles associated with steroido-genesis. Studies in mice demonstrate increased blood testosterone concentration after treatment with polyethylene glycol (PEG)-modified AuNP [86,87]. The presence of AuNPs does not affect the parameters of sperm viability in porcine gametes [88]. However, in human spermatozoa, 50 nm AuNPs have been shown to impact both viability and motility [59]. In a comprehensive toxicological study by Chuang et al. [38], AuNPs were found to substantially modulate the gene expression of 436 genes and protein functions associated with apoptosis and cell cycle progression in mammalian cell lines. Similarly, Ng et al. [44] reported disruption of the expression of 19 genes in human fetal lung fibroblasts due to AuNPs. Changes in gene expression were also observed by Balasubramanian et al. [11] following a single exposure of 0.01 mg/kg to AuNPs. In contrast, AuNPs can also regulate gene expression, aiding disease treatment by silencing genes [89]. Numerous studies indicate that AuNPs induce alterations in cell morphology. For example, exposure of A549 cells to AuNPs for 48 h results in a circular shape due to induced stress [36]. Vetten et al. [43] investigated the effects of AuNPs in an ATP-based assay, which involved the conversion of luciferin to luminescent oxyluciferin with the assistance of ATP. These NPs reduce the luminescence signal, which is particularly notable at higher NPs levels. Several other studies corroborate the decreased ATP levels in treated cells, indicative of mitochondrial dysfunction [90,91]. Additionally, there is consensus among multiple studies on reducing glutathione levels in cells after incubation with AuNP [86,87]. Gao et al. [56] conducted an assessment of the impact of the association between 8-nm AuNPs and glutathione on apoptotic signalling events in human liver cell lines (HL7702 cells). The study revealed an initial reduction in cytosolic GSH levels in HL7702 cells, accompanied by the depolarisation of mitochondrial transmembrane potential and subsequent induction of apoptosis. Tsai et al. [62] used double labelling of propidium iodide and annexin V in conjunction with flow cytometry to elucidate the effects of AuNPs on cell death in osteoblast-like MG63 cells. Patra et al. [36] reported substantial changes in nuclear morphology, including nuclear condensation, indicating cytotoxicity, following exposure of A549 cells to AuNPs. In a hemolysis test by Liu et al. [86,87], polymer-modified AuNPs demonstrated compatibility with human red blood cells, which indicates a dose-dependent toxic potential of SD-AuNPs, accompanied by a significant decline in cell viability and intracellular ROS generation [92]. The growth of AGS, A549, NIH3T3, PK-15, and Vero cells is suppressed in a dose-dependent manner by AuNPs, involving mechanisms such as cell cycle delay and apoptosis induction [38]. Chueh et al. [65] provide evidence of reduced cell growth linked to apoptosis in Vero cells, autophagy in NIH3T3 cells, and DNA damage in MRC-5 cells following AuNPs treatment. AuNPs also decrease breast cell proliferation (MDA-MB-231) [61]. Minimal to no immuno-toxic, cytotoxic, or genotoxic effects are observed in human cells after treatment with AuNPs [58]. Similarly, smaller citrate-stabilized AuNPs show no toxicity to CHO, BEAS-2B, and HEK293 cells, while larger AuNPs exhibit increased toxicity [43]. No cytotoxicity is noted in HeLa cells post-AuNPs treatment [50], nor in A549 and Vero cells treated with AuNP conjugates [34]. Moreover, acute cytotoxicity is not induced in Caco-2 cells by AuNPs [42], and Vero cells exposed to porphyrin-reduced AuNPs exhibit no toxic effects [68]. Functionalized AuNPs do not prompt morphological changes or cell death in tumor ascites and normal peritoneal cells [67]. Silica-coated AuNRs and glucose-capped AuNPs demonstrate no toxic effects in HeLa cells [51,52]. Phosphine-stabilized and thiol-stabilized AuNPs alter gene expression but do not exhibit toxicity in HEK293 cells [47]. These varying outcomes may stem from differences in cell lines, toxicity assessment methods, and the physico-chemical properties of the nanoparticles under investigation. For example, cytotoxicity results may vary depending on the cell line employed; AuNPs with a diameter of 13 nm, capped with citrate, were toxic in human carcinoma lung cell lines but not in human liver carcinoma cell lines at equivalent concentrations [36]. Given these inconsistencies, it is crucial to comprehensively assess the toxicological effects of AuNPs and identify early markers indicative of their health implications.

### 3.2. In Vivo Toxicology Studies of AuNPs

Although there is a considerable body of literature on in vitro studies, there is a limited number of toxicological reports on AuNPs in animal models. Animal models are the preferred system for the toxicological evaluation of new agents, providing crucial information on the potential toxicity of AuNPs [93]. It has become imperative to evaluate the in vivo profile of nanomaterials before considering any therapeutic applications [94] Key in vivo toxicity studies are presented in Table 2.

It is widely accepted that AuNPs accumulate significantly in the liver and spleen, potentially causing further damage to the organism [93]. Gold nanorods (AuNRs), when subcutaneously injected into mice, primarily stayed within the injection site. However, Au ions released into the system resulted in tissue oxidative damage at the injection site [115]. Various researchers have conducted studies to assess the distribution and accumulation of AuNPs in the liver and other organs. Exposure of HT-29 and HepG2 cells and Wistar rats to 10, 30, or 60 nm AuNPs allowed evaluation of their localization and distribution in sub-cellular compartments and tissues, together with other effects [54]. Traces of AuNPs were detected in the liver, intestine, urine, feces, kidney, and spleen. Transmission electron microscopy revealed the presence of particles in colon cells and liver samples. The size of the nanoparticles played a pivotal role in determining differences in biodistribution and excretion routes, with smaller NPs inducing greater harmful effects, as evidenced by DNA damage within the cell nucleus. Ultra-small AuNPs demonstrated superior circulation times and distinct biodistribution compared to larger counterparts, emphasizing the role of physico-chemical properties, including size, shape, and surface coating. Schmid et al. [116] highlighted that ultra-small AuNPs can exhibit cytotoxic properties when stabilisation ligands allow direct access to the Au surface for catalytic activity or direct association with biological molecules. Fraga et al. [117] reported that the surface coating of AuNPs significantly influenced toxicity more than biodistribution. To support this, another study revealed the size-dependent distribution of NPs in rats after exposure to AuNPs at 5.3 μg/rat, with surface charge and size influencing the distribution [118]. Similarly, AuNPs exhibited a size-dependent distribution, with smaller particles showing the highest widespread distribution and the ability to cross the blood–brain barrier. Despite the blood–brain barrier acting as a preventive measure against AuNPs in the central nervous system, ultra-sized particles could cross, reaffirming the size-dependent influence of AuNPs [105,109]. Au has been reported not to be detected in fetal organs but was present in the placenta after exposure to 20 and 50 nm AuNPs, with no apparent toxicity observed in the fetus or placenta [103]. The research found no increase in endocytic vesicles within syncytio-trophoblasts and fetal endothelial cells at the maternal–fetal barrier, indicating a significant involvement of clathrin and caveolin-mediated endocytosis in the placental passage of AuNPs. A similar study involving pregnant C57BL/6 mice showed no transfer of AuNPs across the placental barrier, likely to be due to their inability to penetrate cell membranes through non-endocytic processes [110]. Intraperitoneal injection of AuNPs in rats resulted in significant discrepancies in specific liver enzymes, while those capped with trisodium citrate dihydrate induced mild nephrotoxicity and hepatotoxicity [63,119]. AuNPs, utilized as bio-labels, bio-sensors, and drug carriers, induced hepatotoxicity, cytotoxicity, and toxicity in the spleen and lungs [120,121,122]. Apoptosis and inflammation were observed in liver tissue of mice (BALB/c) following intravenous administration of AuNPs [95,96], particularly targeting the liver and spleen, the primary organs involved in detoxification. Minimal liver damage was noted in mice injected with PEG-coated AuNPs [106]. Moreover, only larger spherical AuNPs were found in the bloodstream, spleen, and liver, whereas smaller particles (~10 nm) were detected in various organs of male WU Wistar rats, including the thymus, lungs, kidneys, testes, heart, and brain [105]. The spleen and liver were identified as the primary organs for nanoparticle accumulation via the reticulo-endothelial system, potentially leading to toxicity [5,123]. Sadauskas et al. [102] observed spherical AuNPs in the liver and macrophages in female mice, with AuNPs of all sizes present in the spleen, liver, and lungs of mice (ddy) [109]. However, hepatocytes showed no signs of toxicity after 24 h of exposure. In male Wistar rats exposed to AuNPs, genes regulating both up and down expressions were noted, with the particles persisting and accumulating in the spleen and liver [11]. Oral, tail vein, and intraperitoneal injection of citrate-coated AuNPs in mice revealed significant toxicity and impacted organ indices [93]. Hence, functionalization and capping have the potential to introduce toxicity to AuNPs. Many toxicological studies involving AuNPs have utilized particles with various capping, conjugating, or stabilizing agents. To enhance their effectiveness in different applications, AuNPs are stabilized, coated, conjugated, or functionalized with various organic moieties, providing a protective layer on the particle’s surface. Most of these molecules exhibit lower cytotoxicity and favorable biodistribution [124,125]. Various levels of toxicity have been associated with the use of certain stabilizing, capping, or conjugating agents for AuNPs, including sodium borohydride, hydrazinium hydroxide, citrate [82], polyelectrolyte poly (allylamine) hydrochloride [126], and CTAB [127]. High-molecular-weight PEG of 5000 Da imparted greater stability to coated AuNPs than low molecular weight PEG (2000 Da) [128]. In particular, high-molecular-weight PEG-stabilized nanoparticles were less toxic [129]. Glutathione emerged as an alternative to PEG in constructing AuNPs for therapeutic purposes due to its biocompatibility and low immunogenicity [123]. A study by Vijayakumar et al. [82] aimed to compare the cytotoxic effects of three stabilizing agents (citrate, starch, and gum Arabic (GA)) on PC-3 and MCF-7 cell lines. They observed that citrate-coated AuNPs exhibited higher cytotoxicity at elevated concentrations compared to starch- and GA-coated AuNPs, which is possibly attributed to the acidic nature of citrate. Citrate-coated AuNPs colloids demonstrated the ability to cross the blood-brain barrier and accumulate in neurons, liver, spleen, and kidneys without observed toxicity [114]. Despite the blood–brain barrier traditionally preventing central nervous system (CNS) access for AuNPs, their crossing, in this case, was linked to the citrate coat. Furthermore, intratracheal exposure to PEG-coated AuNPs accumulated in the liver and spleen, inducing apoptosis and acute inflammation in the mouse liver [96,111].

PEG-coated AuNPs also induced size-dependent cytotoxicity mediated by ROS, with 42.5 and 61.2 nm-sized particles primarily accumulating in the spleen and liver. They underscored that in vitro toxicity was contingent on size and dosage, with higher levels and smaller AuNPs leading to increased cytotoxicity. Furthermore, smaller sizes were found to cause more damage to cells through ROS production. Biodistribution, as highlighted, depended on size and exhibited elements of accumulation and clearance [112]. Histological examinations supported the in vivo activity of AuNPs, particularly in rat liver sections, validating histology as a credible technique for assessing NPs toxicity [130]. Different effects and distributions of AuNPs were observed in histological examinations of the testes, liver, and kidney, with mild changes noted in kidney and liver sections but no effects in the testes [113]. GSH-coated AuNPs were observed to lack kidney toxicity, unlike a study involving clusters protected with tiopronin monolayers (TMPC) at equivalent concentrations. Additionally, Au nanoclusters shielded by GSH and BSA (AuNC) impacted kidney function in mice, resulting in toxicity reactions that resolved within 28 days [107]. This phenomenon may be explained by the kidney’s role in filtering nanoparticles in the renal glomeruli into the urine [110]. Introducing 15 ppm AuNPs into broiler chicken drinking water resulted in evident oxidative damage in blood, histopathological alterations, up-regulation of IL-6, expression of the Nrf2 gene, DNA fragmentation, and a significant decrease in antibody titers against avian influenza and Newcastle disease [97]. In mice, AuNPs were found to induce damage to the neuronal system [101,131], while porphyrin-reduced AuNPs did not exhibit anomalies [68]. However, functionalized AuNPs were observed to accumulate in various brain parts in male CD1 mice [104]. Citrate-capped AuNPs were implicated in causing transmissible mutagenic effects in *Drosophila melanogaster* [99]. Pompa et al. [100] investigated the effects of 15-nm citrate-capped AuNPs in *Drosophila melanogaster*, noting declines in fertility and lifespan, DNA fragments, and overexpression of stress proteins after daily ingestion of 12 μg/g AuNPs. This illustrates how nanoparticles can impact complex biological systems upon introduction. A substantial amount of AuNPs led to declines in mice’s red blood cells and body weight, with oral administration inducing reductions in red blood cells, spleen index, and body weight [93]. A significant proportion of mice died within 21 days after exposure to 8–37 nm naked colloidal AuNPs, experiencing weight and appetite loss [5]. Toxicity studies were also conducted in lower model organisms, such as fish, reporting LC50 values. For instance, the LC50 of HAuCl4 was reported as 2 mg/L after 48 h in *Daphnia magna* [70], 0.64 mg/L [98], and 0.62 mg/L in *Moina macrocopa* [64]. The LC50 was 14.4 mg/L in *Thamnocephalus arcticus* after 96 h [98]. Exposure to AuNPs was highlighted as inducing a dose-dependent response, highlighting the potential hazards posed by nanoparticles [132]. Understanding their characteristics and behavior is crucial to understanding their overall impact.

### 3.3. Toxicity of AuNPs against Immune Cells

Gold nanoparticles may induce effects beyond toxicity, as they can intricately influence cells’ immunological responses [133]. Macrophages, central to orchestrating inflammatory reactions, offer a valuable avenue for conducting preliminary toxicological assessments of nanomaterials because of their interactions with functionalized nanoparticles. Shukla et al. explored the immunogenic effects of gold nanoparticles on RAW264.7 macrophage cells, revealing a remarkable viability exceeding 90% and the absence of an increase in pro-inflammatory cytokines TNF-a and IL-1b, even after 48 h of exposure to gold nanoparticles up to 100 nm [134]. An intriguing parallel investigation focused on PEG-coated gold nanoparticles, which exhibited a notable absence of cytotoxic effects. Nevertheless, these AuNPs exhibited an unforeseen capacity to amplify the immune response prompted by external stimuli, like LPS. In particular, gold nanoparticles coated with PEG augmented the expression of inducible nitric oxide synthase and the production of interleukin-6 (IL-6) in RAW264.7 cells induced by LPS. This enhancement was associated with the activation of the p38 mitogen-activated protein kinases (p38 MAPK) and nuclear factor kappa B pathways, as per molecular mechanisms [135]. Developing an airway epithelial model, including macrophages and human monocyte-derived dendritic cells, contributed to understanding the phenomenon. Surprisingly, no inflammatory responses were observed when these cells were exposed to 15 nm gold nanoparticles, suggesting a lack of immune reaction [136]. However, when these gold nanoparticles were coated with peptides, a distinct immune response was induced after recognition by primary murine macrophages, marked by the secretion of immune-related molecules, including IL-6, IL-1b, and TNF-a. This observation suggests that coating gold nanoparticles with peptides can significantly enhance their immune effects, showing promising potential for developing hybrid nanoparticles tailored to modulate immune responses in addressing allergies, cancer, and auto-immune disorders [137]. The impact of plasma proteins adsorbed onto nanoparticles upon entering the bloodstream has garnered attention due to its potential to interfere with the presentation of other ligands attached to the particles. Evaluating CTAB- and PEG-coated gold nanorods revealed their potential adverse effects on the human immune system, particularly regarding allergy induction. Specifically, CTAB-coated GNRs were found to release a higher number of allergic mediators, including histamine from human basophil KU812 cells, and induced more apoptosis compared to their PEG-coated counterparts in KU812 cells. This emphasizes the crucial role of surface material in triggering allergic reactions [106,138]. Furthermore, an intriguing discovery emerged, indicating that the immunological response of macrophages intensified with decreasing gold nanoparticle size [6]. As immune effector cells, macrophages exhibited distinctive signs of cell activation, including spread morphology and larger sizes when exposed to gold nanoparticles. Investigation of the immunological response also entered the realm of gene expressions, revealing upregulation of pro-inflammatory genes, such as IL-1, IL-6, and TNF-a [6]. These nuanced findings deepen our understanding of the intricate interplay between gold nanoparticles and the immune system, offering valuable insights for future research and the potential development of novel therapeutic strategies.

### 3.4. Toxicity of AuNPs against Normal Human Cell Lines

#### 3.4.1. Nervous System

The toxicity of AuNPs toward human nerve cells in vitro is very poorly understood, and there are few reports on this subject in the scientific literature. Research by Trickler et al. regarding the effect of gold nanoparticles on porcine brain micro-vascular endothelial cells (pBMECs) in the context of the secretion of pro-inflammatory mediators and the impact on the blood–brain barrier (BBB) in vitro showed that, unlike AgNPs and oxide copper (CuNP), exposure to gold nanoparticles (3 and 5 nm sizes) did not induce significant secretion of pro-inflammatory mediators (IL-1b, TNFα, PGE2) or negatively affect the integrity of the BBB. The results suggest that the composition and size of the nanoparticles may significantly impact the pro-inflammatory response, which may impact the integrity of the BBB, with gold nanoparticles exhibiting less neurotoxicity than silver and copper oxide nanoparticles [139]. The study by Senut et al. (2016) provides critical insights into the neurotoxic effects of AuNPs of different sizes on human embryonic stem cells (hESCs) and their neural derivatives, emphasizing the nuanced impact of nanoparticle size on neurodevelopmental processes. The research demonstrates that AuNPs, especially those with a core size of 1.5 nm, exhibit pronounced neurotoxic effects on hESCs and their derived neural progenitor cells. Exposure to these nanoparticles resulted in significant cell death and disruption of neural differentiation, indicating a potential risk to the integrity and functionality of developing neural tissues. These findings are particularly concerning given the increasing interest in using nanoparticles for brain-targeted drug delivery systems, diagnostic tools, and other neurotherapeutic applications. The observed neurotoxicity could be attributed to several mechanisms. The small size of the 1.5 nm AuNPs allows for easy penetration through cellular membranes, facilitating direct interactions with intracellular components crucial for neural differentiation and survival. The disruption of embryoid body formation and detachment of hESCs suggests that nanoparticle exposure compromises cell adhesion and intercellular communication, essential processes for neural tissue development. Furthermore, the study findings on altered DNA methylation patterns in response to 4 nm AuNPs exposure hint at epigenetic modifications as another layer of complexity in nanoparticle-induced neurotoxicity [140]. Given the critical findings of Senut et al. (2016), future research should focus on delineating the precise mechanisms of AuNPs-induced neurotoxicity, exploring the role of surface modifications in modulating these effects and establishing safety guidelines for using nanoparticles in neurobiology. Investigating the long-term impacts of nanoparticle exposure on neural function and development is also essential to ensure the safe integration of nanotechnologies into therapeutic strategies.

#### 3.4.2. Digestive System

In the discourse on the toxicity of nanoparticles toward the digestive system, there have been very few in vitro studies on human cell lines. The study by Aueviriyavit et al. (2014) investigated the cytotoxic effects of gold nanoparticles on Caco-2 cells, a model of the human intestinal epithelium, to understand their potential impact on human health [42]. Unlike silver nanoparticles (AgNPs), which have been shown to induce significant cytotoxicity and oxidative stress in various cell lines [141,142,143], AuNPs demonstrated a markedly different interaction with Caco-2 cells. These findings suggest that AuNPs are internalised by Caco-2 cells without causing significant cytotoxic effects or oxidative stress, indicating a potentially safer profile for biomedical applications. The lack of Nrf2/HO-1 pathway in response to AuNPs exposure further supports the hypothesis that these nanoparticles do not provoke a substantial response to oxidative stress in Caco-2 cells. This starkly contrasts with the effects observed with AgNPs, where antioxidant defence mechanisms were strongly activated. This distinction could be attributed to the inherent physico-chemical properties of AuNPs, including their size, shape, and surface chemistry, which can influence their cellular interactions and toxicity profiles [42].

The study by Yao et al. (2015) serves as a significant reference point, mainly due to its focus on the impact of gold nanoparticles on intestinal epithelial cells. Conducted in a model intestinal epithelial cell line, this research elucidates the nuanced interactions between AuNPs and the cells lining the GI tract, offering insights into the potential health implications of nanoparticle ingestion. The study’s findings highlight a critical aspect of nanoparticle toxicity: the size-dependent penetration and accumulation of AuNPs in intestinal epithelial cells. Research revealed that the intestinal epithelium more readily absorbed smaller AuNPs (15 nm) than their larger counterparts, which could significantly influence the biodistribution and bioaccumulation of these particles within the GI system. Interestingly, smaller nanoparticles exhibited decreased accumulation within cells despite increased penetration. This inverse relationship between size, absorption, and accumulation underscores the complexity of nanoparticle–cell interactions and suggests that smaller nanoparticles might cross the epithelial barrier more efficiently, potentially leading to systemic exposure. In addition, the study on the intestinal epithelial cell line model provided crucial evidence of the cytotoxic effects of the accumulation of AuNPs. Depolarisation of mitochondria membranes indicates mitochondrial dysfunction, a marker of cellular stress, and a potential initiator of cell death. Given the central role of mitochondria in energy production and cell survival, this finding is particularly concerning, highlighting the potential for nanoparticle-induced toxicity in the gastro-intestinal tract. In conclusion, the toxicity of nanoparticles toward the gastro-intestinal system, as demonstrated by research on a model intestinal epithelial cell line, raises important considerations for the safety assessment and regulatory oversight of nanomaterials. Understanding the size-dependent behaviours of nanoparticles within the gastro-intestinal tract is crucial to predicting their biological impacts and designing safer nanotechnology-based applications [144].

#### 3.4.3. Respiratory System

Surprisingly, there is little data on the toxicity of gold nanoparticles on the respiratory system in a human cell line, given the fact that the lung is one of the primary pathways through which nanoparticles enter the body, making it a probable location for the accumulation of this particle [145]. The human lung fibroblast cell line MRC-5 was exposed to a concentration of 1 nM AuNPs in a study by Li et al. [64]. Hydroperoxide levels were higher in treated cells than in the control group, similar to the number of proteins modified by malondialdehyde, indicating that AuNPs could induce oxidative stress in lung cells. The formation of autophagosomes was also observed, with a significantly increased expression of some autophagy-related proteins, such as MAP-LC3-II and ATG 7. Expression of other autophagy-related proteins (ATG5, BECN1, ATG12) was also observed but was insignificant compared to the control. This finding confirmed previous studies [76], suggesting that AuNPs could cause oxidative stress damage in lung fibroblasts.

#### 3.4.4. Cardio-Vascular System

Although the cardio-vascular system plays a crucial role in the distribution of particles, there is a lack of data regarding the toxicity of AuNPs in normal human cell lines. Most of the available data include in vivo studies in rats and mice. In a study conducted by Abdelhalim [146], the rats treated with 10 and 20 nm gold nanoparticles for 3 or 7 days exhibited congested heart muscle with dilated blood vessels, scattered red blood cells outside the vessels, muscle hyalinosis, disrupted muscle fascicles, and a dense focus of inflammatory cells infiltrated by small lymphocytes and few plasma cells. On the contrary, rats treated with 50-nm AuNPs for the same duration showed normal-looking heart muscle with regular muscle direction and fascicles, along with only a few scattered small lymphocytes. Yang et al. [147] assessed the chronic cardiac toxicity of polyethylene glycol (PEG) coated AuNPs of varying sizes (ranging from 10 to 50 nm) in mice for 14 days. Mice were euthanised at 2, 4, or 12 weeks post-initial injection for analysis. The accumulation of AuNPs in the heart and their impact on cardiac function, structure, fibrosis, and inflammation were evaluated. Smaller AuNPs showed greater accumulation and faster elimination. Although no AuNPs sizes affected cardiac systolic function, mice injected with 10 nm PEG-AuNPs exhibited significant increases in left ventricular dimensions, mass, and heart weight/body weight ratio after two weeks, indicating reversible cardiac hypertrophy. An example of an in vitro human cell line of the vascular system is provided by Uchiyama et al. [148]. The study did not show significant cytotoxicity on the HUVEC cell line. The bioconjugates of AuNPs, despite being internalised and detected in the cytoplasm, did not cause lysis of human erythrocytes, apoptosis, or necrosis of human leukocytes or endothelial cells in vitro. In this study, in vivo alterations in the micro-vascular system were also evaluated in Wistar rats.

#### 3.4.5. Urinary System

Enea et al. investigated the potentially toxic effects of AuNPs on human kidney cells using the HK-2 cell line. AuNPs of varying sizes (13 nm and 60 nm), shapes (spheres and stars), and coatings (11-mercaptoundecanoic acid or sodium citrate) were synthesised and evaluated for their toxicity [149]. The evaluation included viability, lysosomal integrity, mitochondrial membrane potential, reactive oxygen/nitrogen species production, intracellular glutathione levels, ATP production and apoptosis. The results indicate that smaller 13 nm nanospheres, particularly those coated with MUA, exhibit the highest toxicity, affecting mitochondrial function and inducing programmed cell death. In contrast, larger 60 nm AuNPs appear to be less toxic. Another study by Zhao et al. aimed to investigate the effects and mechanisms of gold nanoparticles of different sizes in HK-2 and 786-0 cells [150]. HK-2 and 786-0 cells were treated with 5 nm and 200 nm AuNPs at 1 and 10 μg/mL concentrations. Various cellular parameters were analyzed, including cell viability, intra-cellular reactive oxygen species levels, apoptosis, autophagy, and related signalling pathways. The results showed that in HK-2 cells, AuNPs reduced the activity of Akt and mTOR while increasing the expression of LC3 II. In 786-0 cells, AuNPs upregulated the activity of p38, leading to increased caspase three activity and the initiation of apoptosis. In conclusion, 5 nm and 200 nm AuNPs at 10 μg/mL demonstrated anti-tumor effects by inducing apoptosis and inhibiting cell proliferation.

#### 3.4.6. Sensory Organs

The toxicity of gold nanoparticles, synthesised with *Vitis vinifera* seed extract, was tested against keratinocyte cell line (HaCaT) and human epidermoid skin cancer cell line (A431) in a study conducted by Nirmala et al. [151]. AuNPs did not show cytotoxicity against HaCaT cells. However, cytotoxicity against cancer cells was associated with increased levels of reactive oxygen species, induction of apoptosis, and morphological changes.

#### 3.4.7. Reproductive System

A study by Wiwanitkit et al. aimed to assess the toxicity of gold nanoparticles on human sperm [152]. The nanoparticles were synthesised using the Turkevich method and mixed with semen. The results showed that 25% of the sperm lost mobility after exposure to AuNPs, with evidence of nanoparticle penetration into the heads and tails of the sperm.

#### 3.4.8. Human Cell Lines’ Toxicity Evaluation

Evaluating the complex organ toxicity of gold nanoparticles requires a thorough evaluation of their potential effects on various organs within the body using suitable cell lines. A summary of the considered studies on cell lines is provided in Table 3.

### 3.5. Unfavourable Effects of AuNPs

Gold nanoparticles have been used for diagnostic and therapeutic purposes; however, recent research shows that their cytotoxicity may differ depending on the cell type and degree of AuNPs aggregation. The concentration of gold nanoparticles is also important here [40,83,153]. Studies show that concentrations above 20 nM significantly affect human embryonic neural precursor cell proliferation without causing apoptotic death [154]. Chueh et al. demonstrated that different mammal cell lines have various sensitivities to the cytotoxic effects of AuNPs; for example, Vero cells (African green monkey kidney) exhibit the highest sensitivity, while PK-15 cells (porcine kidney) exhibit the lowest sensitivity [65]. The findings presented in this report indicate that AuNPs modulates physiological processes in various cell types through diverse pathways, depending on the cellular context and genetic composition. Nanoparticles can produce two distinct types of toxicity in human and animal cells. One type is cytotoxicity, characterised by inhibiting enzyme activity, leading to acute and immediate effects, such as apoptosis induction in Vero cells by AuNPs. The other type of toxicity is genotoxicity, which involves harmful actions on cellular genetic material (DNA or RNA) and is commonly associated with the development of cancers and genetic disorders. Cells have also exhibited high susceptibility to AuNPs toxicity measuring 1.4–1.5 nm in diameter, specifically Au55 nanoparticles [4,155]. Examination revealed that clusters of human embryonic stem cells (hESCs) treated with 1.5 nm MSA-capped AuNPs exhibited loss of cohesion, rounding, and detachment, indicating an ongoing cell death process. Furthermore, hESCs exposed to 1.5 nm AuNPs did not aggregate to form embryoid bodies (EB) but disintegrated into individual cells rapidly within 48 h of exposure [140].

### 3.6. Organ Toxicity of AuNPs

AuNPs can enter the body by ingestion or intravenous administration. Once they enter the body, they can affect various parameters. The investigation by Lasagna et al. focused on examining the bioaccumulation and biodistribution of AuNPs in mice after repeated administration [114]. Their study revealed that repeated administration of AuNPs did not result in any mortality or signs of toxicity. This conclusion was drawn based on various factors, including animal behaviour, tissue morphology, serum biochemistry, hematological analysis, and histopathological examination. However, Chen et al. presented contrasting findings, demonstrating that AuNPs invasion can have lethal effects in mice [5]. Furthermore, Cho et al. conducted a separate study that indicated that 13 nm gold nanoparticles coated with PEG exhibited acute toxicity [96]. According to Zhang et al., the oral and intraperitoneal routes exhibited the highest toxicity among the three administration routes for gold nanoparticles in animals. In contrast, the intravenous route showed the lowest toxicity [93]. The available literature presents various methods and conclusions in both in vitro and in vivo studies. The toxicity effects of AuNPs vary significantly depending on factors such as particle size, pH, charge, conjugation, route of administration, and dosage.

Due to their minute size and expansive surface area, AuNPs exhibit increased activity and occasionally display unforeseen consequences when interacting with biological systems. To investigate the toxicity of acute and chronic exposure in an in vivo animal model, Sengupta et al. established four distinct groups of mice [156]. The initial group served as the control, receiving an intravenous injection of 200 μL of 0.9% saline solution, while groups II to IV were administered intravenous injections of 200 μL of AuNPs solution at concentrations of 1 mg/kg, 2 mg/kg, and 10 mg/kg, respectively. After injections, urine samples were collected after 24 h, and blood samples were obtained at intervals of 6, 12, 24, 48, and 72 h. A control group and two experimental groups were established for chronic evaluation of the impact of AuNPs on mice organs, receiving intravenous injections of 200 μL of AuNPs solution at concentrations of 1 mg/kg and 2 mg/kg. Blood and urine samples were collected for analysis after 15, 30, 60, and 90 days. The animals were sacrificed after 30, 60, and 90 days, respectively, to perform examinations on internal organs such as the lungs, kidneys, liver, and spleen. After a single dose of exposure to AuNPs, also known as an acute study, the treated group was observed to experience an increase in total white blood cell (WBC) count. This increase in the WBC count indicates that AuNPs have the potential to affect the immune system of the body. The differential count analysis revealed a significant 15–20% increase in blood lymphocyte counts, suggesting that AuNPs can trigger an immune response. Furthermore, the study indicates that nanoparticles have acute toxic effects on the animal system. Hematological studies have also shown a dose-dependent increase in the percentage of hemoglobin and the count of red blood cells (RBC). These findings suggest an active interaction between AuNPs and blood WBC, hemoglobin, and RBC, which depends on the dosage. Furthermore, the presence of blood in the urine of the AuNPs treated group (at a dosage of 10 mg/kg) indicates a detrimental effect on the renal system at this particular dosage. The observations highlight that AuNP-induced hematological changes are significantly more pronounced at higher dosage levels (2 mg/kg and 10 mg/kg), possibly due to hematopoietic stimulation. However, no significant changes were observed in blood hemoglobin and RBC at a lower dose (1 mg/kg). The significant increase in WBC count at the low dose level (1 mg/kg) can be considered an important aspect of the WBC stimulation pathway, which is maintained at the other two higher dose levels (2 mg/kg and 10 mg/kg). Chronic studies conducted at two dose levels (1 mg/kg and 2 mg/kg) have demonstrated a dose-dependent effect of AuNPs on the animal system. The acute symptoms and eventual death of mice that receive a dose of 2 mg/kg AuNPs indicate that injected AuNPs may cause damage to the main organs. Furthermore, noticeable physical changes, such as changes in fur colour and texture, as well as skin flaccidity, have been observed in the AuNPs treated groups (at doses of 1 mg/kg and 2 mg/kg), indicating the adverse effects of prolonged exposure to AuNPs on the body system. Most mice in the group who received a dose of 2 mg/kg died within 30 days of the treatment schedule, further emphasizing the high toxicity of AuNPs on the body system after multiple exposures. Despite the absence of any noticeable changes in serum biochemical parameters (urea, creatinine, LDH, and GPT), the tissue histology of the AuNP-treated groups exhibited significant alterations in tissue histopathology after 30, 60 and 90 days at a concentration of 1 mg/kg and after 30 days at a concentration of 2 mg/kg. These pathological changes indicate the harmful impact of chronic exposure to AuNPs. On the contrary, the research conducted by Sengupta et al. [156] contradicts the findings of Lasagna et al., suggesting that AuNPs within the 50-nm range can cause detrimental physiological changes in mice after acute and chronic treatment [114]. In particular, acute exposure to AuNPs resulted in severe detrimental changes in liver morphology, as observed by tissue histology. The findings of acute and chronic examinations unequivocally demonstrate that AuNP-induced toxicity becomes apparent when administered at high doses (2 and 10 mg/kg). In contrast, when administered at a low dose level (1 mg/kg), there is a negligible impact. Furthermore, the observed changes at the low dose level (1 mg/kg) were only discernible after prolonged and repeated exposure.

Previous research on the toxicity of AuNPs in vivo has indicated that the size of the particles plays a crucial role. Specifically, smaller AuNPs measuring 5 and 10 nm induced significant histopathological changes in mice livers, while larger 20 and 50 nm particles had only minor effects [157,158]. Interestingly, a separate study revealed that intraperitoneal injection of AuNPs ranging from 8 to 35 nm resulted in severe illness and high mortality, a phenomenon not observed with smaller particles (3–5 nm) or larger particles (50–100 nm) particles [5]. This trend was also observed in toxicity studies involving oral administration of AuNPs, suggesting that factors such as surface coating, dosage, exposure route, duration, and species can also influence the in vivo toxicity of AuNPs. A study by Sun et al. utilized a colloidal solution of gold nanoparticles to examine the effects of oral administration of AuNPs on mice [159]. Mice were categorized into three groups: low-dose (LD), medium-dose (MD), and high-dose (HD). The LD group received 0.2 mg/kg of colloidal AuNPs daily, the MD group received 2 mg/kg, and the HD group received 20 mg/kg. A control group was administered ddH_2_O. The livers, spleens, kidneys, lungs, brains, and hearts of the mice were collected, weighed, and subjected to thorough gross necropsy. Throughout the experimental period, no mortality or abnormal clinical signs were observed in either male or female mice, including changes in skin and fur conditions, eye appearance, bowel movements, breathing patterns, activity levels, or tremors. Hematological parameters in both male and female mice in the AuNP-treated groups remained within normal ranges, except for platelet indices in female mice from the highest dose group. Female mice receiving 20 mg/kg AuNPs showed significantly higher platelet count (PLT), mean platelet volume (MPV), plateletcrit value (PCT), and platelet distribution width (PDW) compared to the control group. This suggests that high doses of oral AuNPs can affect platelet and coagulation function in female mice. Furthermore, a slight but significant decrease in serum calcium levels was observed in female mice administered AuNPs at 2 and 20 mg/kg, although these values remained within the normal range. Male mice administered AuNPs at doses of 0.2 and/or 2 mg/kg exhibited significantly elevated serum levels of glutamyl pyruvic transaminase (GPT) and alkaline phosphatase (ALP), indicating liver dysfunction, while this effect was not observed at 20 mg/kg. However, no histopathological changes were observed in the livers of male mice treated with AuNPs. Conversely, serum levels of GTP, ALP, and total bilirubin (T-BIL) did not differ in female mice treated with AuNPs. Interestingly, male mice showed a slight but significant increase in blood urea nitrogen (BUN) levels, while female mice exhibited decreased BUN levels after oral administration of 2 mg/kg AuNPs. In another study by Zhang et al., male ICR mice treated with citrate-coated AuNPs with a size of 13.5 nm at a dose of 2.2 mg/kg for 14 days experienced a significant reduction in body weight and organ indices, alongside an increase in thymus and spleen index [93]. However, Sun et al. found that male ICR mice treated with citrate-coated AuNPs measuring 53 nm at 2 mg/kg for 90 days did not exhibit any abnormal effects [159]. Previous investigations [5,105,109,160] have indicated that AuNPs with smaller particle sizes (<50 nm) tend to display enhanced tissue distribution and accumulation compared to larger particles (50–250 nm) when administered intraperitoneally or intravenously.

### 3.7. Size and Shape Impact of AuNPs on Toxicity

According to Li et al. [112], the distribution pattern of AuNPs varied depending on their size. The larger AuNPs, measuring 42.5 and 61.2 nm, were predominantly found in the liver and spleen. Slowly, these particles were eliminated from the body, with some remnants still present after 90 days. On the other hand, the smaller AuNPs, measuring 6.2 and 24.3 nm, exhibited a broader biodistribution across multiple organs. These smaller particles were excreted relatively faster compared to their larger counterparts. Ding et al. [161] conducted a study investigating the impact of various design parameters on the uptake and removal mechanisms of five different types of gold nanoparticles with different sizes and shapes. These included spherical NPs with average diameters of 15 nm (NP1), 45 nm (NP2), and 80 nm (NP3), as well as nanorods (NR) measuring 33 × 10 nm and nano-stars (NS) with an average diameter of 15 nm. The researchers prepared these nanoparticles and examined their effects. The study results revealed that the spherical NPs exhibited the lowest toxicity compared to NR and NS. Even at a high concentration of 300 mg/L for 24 h, the cell viability remained above 80% when exposed to the three spherical NPs. Furthermore, the toxicity of the spherical NPs demonstrated a size-dependent trend, with toxicity decreasing as the size increased. In contrast, the toxicity of NS and NR was significantly higher than that of the spherical Au NPs. The safe concentration for NR was less than 1 mg/L, while for NS, it was 10 mg/L for 24 h. This difference in toxicity can be attributed to the initial numbers of NPs with different sizes and shapes, which varied significantly under the same original concentration. The study also highlighted the crucial role of different shapes and sizes in the uptake of nanoparticles. Among the five types of NPs, NP3 exhibited the highest uptake ratio, followed by NR. It was observed that the NPs tended to agglomerate and, due to the larger size and weight of agglomerated NP3, its sedimentation rate was much faster compared to other NPs. This sedimentation process influenced cellular uptake, with more NP3 reaching the cell surface and resulting in a higher absorption ratio of nanoparticles on the cell surface. Consequently, this directly increased the amount and rate of nanoparticle uptake. Furthermore, the study indicated that cells were likelier to take cationic NPs. NR, which exhibited a positive charge, showed increased uptake due to electrostatic adsorption between the NR and the cell membrane. On the other hand, NP1, NP2, and NS displayed a negative surface charge, resulting in lower uptake ratios compared to NR. The findings suggest that the uptake of nanoparticles by cells is restricted. A correlation exists between the concentration of nanoparticles and the amount taken up by cells until saturation is reached. Once saturation is achieved, the cellular uptake remains constant, despite further increases in concentration. Other shapes, such old nanocluster, that are generated from gold nanoparticles by significant quantization occurring to the conduction band, leading to altered physical and chemical properties [162]. In study conducted by Zhang et al., it was shown that GSH-protected gold nanoclusters have high renal clearance and can decrease toxicity, while BSA-protected gold nanoclusters can accumulate in liver and spleen, potentially causing irreparable toxicity responses [107].

### 3.8. Toxicity Mechanisms

#### 3.8.1. Mechanism Related to Oxidative Stress

Reactive oxygen species (ROS) formation is considered one of the toxicity mechanisms of nanoparticles that could lead to inhibition of antioxidants and oxidative stress, potentially causing inflammation and damage to molecules and cell membranes [163]. Further oxidative stress can damage DNA, leading to cell activation of cell death pathways [164]. In a study conducted by Ozcicek et al., it was proven that increasing AuNPs concentrations (ranging from 1 µg/mL to 100 µg/mL) correlated with increased ROS generation [165]. In the same study, cell viability levels were measured to be not less than 80%, similar to those of apoptotic cells. It was also indicated that smaller particles were more toxic, and coating nanoparticles with PEI or PEG reduced the apoptotic effects of AuNPs. Treating HeLa cells with gold nanoparticles, especially those of 1.4 nm diameter, coated with triphenylphosphine mono-sulfate, led to ROS generation, which could trigger the necrosis death pathway. Incubation of AuNPs with antioxidant agents (particularly thiol-containing) could increase cell survivability [4]. Oxidative stress caused by various diameters (30 nm, 50 nm, and 90 nm) in human leukemia (HL-60) and hepatoma (HepG2) cell lines was tested in a study conducted by Mateo et al. [55]. GSH levels decreased in both cell lines, indicating an increase in the production of reactive oxygen species, contributing to the cytotoxicity. Gold nanoparticles may cause oxidative stress and damage to biological molecules due to changes in antioxidant enzyme activities in healthy (HEK 293 T) and cancer (A375 and A594) cell lines, as shown in a study by Dvorakova et al. [166]. In another study conducted by Bin-Jumah et al. [162], cytotoxicity of AuNPs was dose-dependent, confirmed by two methods in two cell lines—CHANG (normal) and HuH-7 (liver cancer). The nanoparticles caused an increase in intracellular reactive oxygen species and lipid peroxide levels while decreasing total glutathione and mitochondrial membrane potential in both cell types, with the effects becoming stronger with increasing doses. N-acetyl-L-cysteine effectively suppressed the generation of reactive oxygen species in both cell lines following exposure to the nanoparticles. Both cell lines exhibited DNA damage, with liver cancer cells showing slightly higher sensitivity. In summary, the nanoparticles induced cytotoxicity and apoptosis primarily through oxidative stress mechanisms.

#### 3.8.2. Mechanism Related to Non-Oxidative Stress

The evaluation of AuNPs toxicity includes underlying potential pathways, such as genotoxicity, ROS generation, mitochondrial damage, cell death pathways, toxic material leakage, the interaction of endocrine disruption with molecules, or change in cell morphology [167]. While oxidative stress is a prominent mechanism of nanoparticle toxicity, it is essential to recognise that gold nanoparticles can also induce toxicity through various non-oxidative pathways. The main cytotoxicity of gold nanoparticles is caused by mitochondrial toxicity, which disrupts various metabolic pathways and affects amino acid synthesis, as shown in a study by Ji et al. [168].

A key factor contributing to toxicity can be attributed to functional groups coated within the structure of nanoparticles. In a study conducted by Goodman et al., it was shown that cationic gold nanoparticles exhibit moderate toxicity, while anionic particles are non-toxic [169]. This suggests that electrostatic binding may be a potential mechanism underlying the toxicity of cationic nanoparticles.

In a study by Selim and Hendi, the biocompatibility of gold nanoparticles in human breast epithelial MCF-7 cells was assessed, focussing on cytotoxicity and induction of apoptosis [170]. There was a significant increase in p53, bax, caspase-3, and caspase-9 mRNA expression, while anti-apoptotic bcl-2 expression was down-regulated. This study demonstrated that gold nanoparticles can induce apoptosis in MCF-7 cells through the p53, bax/bcl-2, and caspase pathways. In another study by Zhou et al., gold nanoparticles influenced autophagy in a shape-dependent manner, with nanospheres inducing more autophagosome accumulation compared to nanorods, likely to be due to differences in cellular uptake [171]. As presented in another study by Bucchianico et al., differently sized gold nanoparticles can induce cytotoxicity and genotoxicity through apoptosis, aneuploidy, and DNA oxidation in human primary lymphocytes and murine macrophages [172].

The induction of DNA damage as a potential non-oxidative stress mechanism has been demonstrated as a mechanism of toxicity of gold nanoparticles in vivo in Wistar rats, as shown by Cardoso et al. [173]. Furthermore, this effect was observed when the nanoparticles were exposed to therapeutic X-rays, as demonstrated in a study by Huwaidi et al. [174]. DNA damage can also be induced without ROS, as demonstrated in a study by Abdelhady et al. [175].

**Figure 1:**
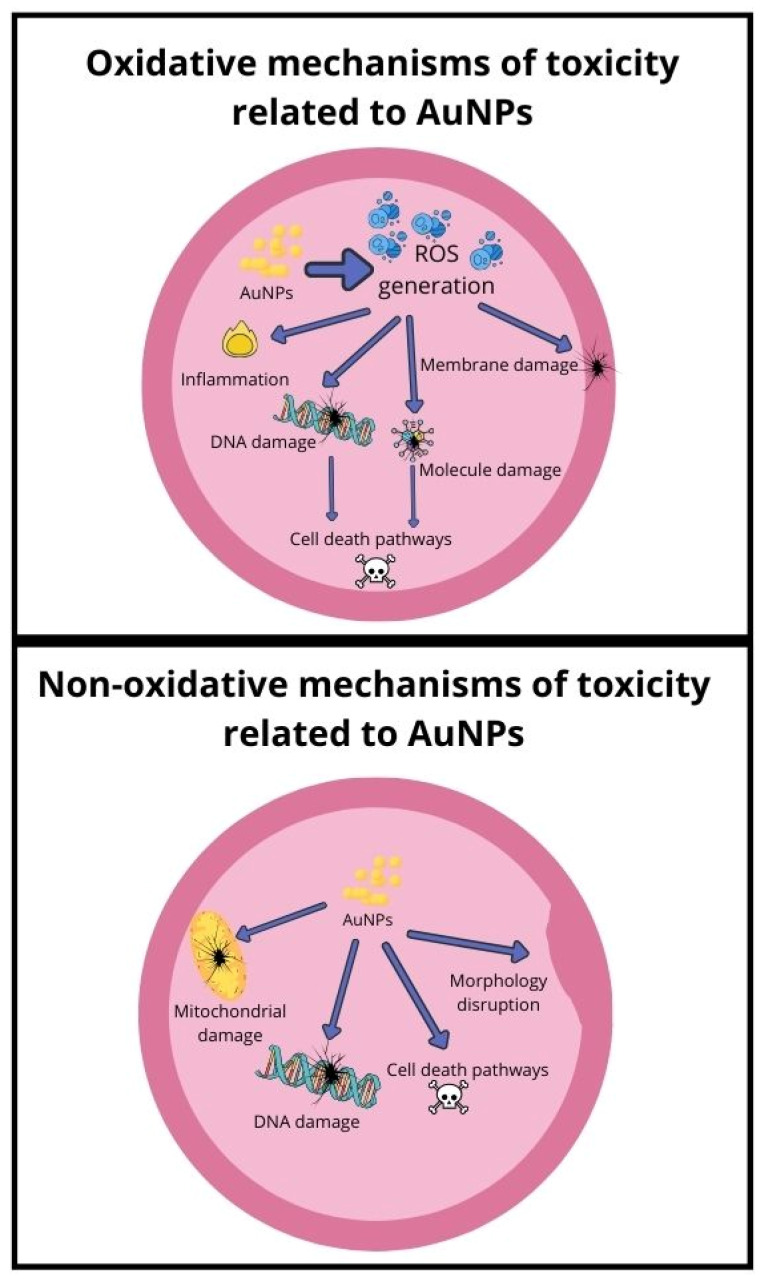
Diagram representing potential oxidative and non-oxidative toxicity mechanisms related to AuNPs.

Diagram representing potential toxicity related to both oxidative and non-oxidative mechanisms is presented in Figure 1. 

## 4. Safety Assessment of Gold Nanoparticles in Cosmetic Products

The current European Union document on safety assessment for using raw materials containing gold nanoparticles in cosmetic products was elaborated according to the opinion issued by the Scientific Committee on Consumer Safety (SCCS)—opinion SCCS/1629/21 [176]. This opinion specifically addresses the use of gold (nano), Colloidal Gold (nano), gold thylamino hyaluronic acid (nano), acetyl heptapeptide-9, and colloidal gold (nano) in cosmetic formulations. The SCCS, an expert committee commissioned by the European Commission, assesses the health and safety risks of non-food consumer products, including cosmetic ingredients. The opinion SCCS/1629/21 provides a comprehensive risk assessment based on available scientific evidence on nanoparticle forms of gold used in cosmetics, highlighting their potential impacts on consumer health and safety. This document is pivotal in guiding regulatory decisions and ensuring the safe use of nanomaterials in cosmetic products within the EU market.

The preparation of this document was based on the fact that the Commission’s services processed notifications (*n* = 237) submitted through the Cosmetic Product Notification Portal (CPNP) according to Article 16 of the Cosmetics Regulation concerning cosmetic products that incorporate gold (with 68 notifications) and colloidal gold (with 169 notifications) in their nano forms, identified by CAS Number 7440-57-5 and EC Number 231-165-9, as detailed in the provided list. In the CosIng database, Gold is catalogued as a colourant (CI 77480) under the designation IV/133 in Annex IV of the Cosmetic Regulation (EC) No. 1223/2009 without specific mention of its nanoform. Similarly, colloidal gold is listed for its antimicrobial and skin conditioning properties without referencing its nano-state. It should be noted that the notifications indicate that both gold and colloidal gold in their nanoforms are incorporated into leave-on skin cosmetics in various concentrations and with distinct specifications, as detailed in the provided documentation. Furthermore, the Commission services received notifications (*n* = 11) through CPNP, as required by Article 16 of the Cosmetics Regulation, for cosmetic products containing gold thylamino hyaluronic acid in nanoform (CAS No. 1360157-34-1, without available CE number), as enumerated in the provided list. Gold Thioethyl-amino Hyaluronic Acid, not specified in its nano form, is recognised in the CosIng database for its role in “skin conditioning” and is outside the specific regulations of the Cosmetic Regulation (EC) No 1223/2009. Based on the notifications submitted, this component was used in topical leave-on skincare cosmetics in varying concentrations and with different specifications.

The Scientific Committee on Consumer Safety (SCCS) has articulated a final stance on the safety of gold nanoparticles in cosmetic products, underscored by a rigorous demand for comprehensive data and information. This stance is predicated on the necessity for detailed characterisation of the (nano) gold, (nano) colloidal gold and (nano) surface-modified gold materials, including their chemical properties, particle size, solubility, surface characteristics, and potential for systemic uptake and toxicity. The SCCS has underscored the importance of assessing acute toxicity, irritation/sensitisation, mutagenicity/genotoxicity, reproductive toxicity and carcinogenicity, especially if there is a significant indication of systemic exposure.

However, SCCS has encountered a critical impasse due to the lack or insufficiency of essential information required for a conclusive safety assessment of these nanomaterials. The predominantly available information pertains to the general properties of gold rather than its nano-formulations, rendering it inadequate for evaluating the safety of the specific nanomaterials under consideration. The lack of comprehensive study reports further exacerbates the challenge of determining the relevance of the existing data for the nano-forms.

The potential systemic uptake of gold nanoparticles is particularly concerning, as the scientific literature suggests, as it could lead to accumulation in vital organs such as the liver and spleen. Furthermore, there is an indication of possible mutagenic/genotoxic effects, necessitating a more in-depth safety evaluation of these nanomaterials within the context of their use in cosmetics.

In light of these considerations, and despite the withdrawal of notifications for certain surface-modified gold materials, like Acetyl heptapeptide-9 Colloidal gold (nano), leaving Gold Thioethyl-amino Hyaluronic Acid as the primary material of interest, the SCCS maintains a position of concern. The committee concludes that, in the absence of comprehensive and relevant data, the use of gold (nano), colloidal gold (nano), and surface-modified gold (nano) materials in cosmetic products could potentially pose a risk to consumer safety.

## 5. Green Toxicology of Gold Nanoparticles

### 5.1. Green Synthesis of Gold Nanoparticles

**Figure 2:**
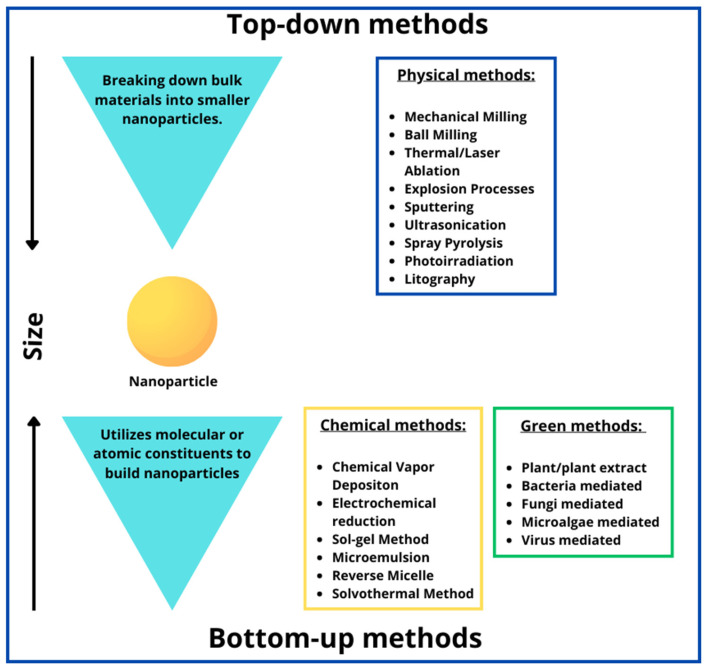
Methods for nanoparticle synthesis based on [184,185].

The approach to green synthesis of gold nanoparticles is generally considered simple but non-facile, eco-friendly, cost-effective, and inexpensive [177]. Like chemical methods, this strategy is classified as a bottom-up technique, unlike physical methods, which are considered top-down methods [178]. The reducing agent, derived from a biological source, provides electrons responsible for reducing positively charged ions (Au^3+^) to Au^0^ neutral atoms [179]. Numerous compounds, including sugars, phenolic compounds, flavonoids, and proteins, exhibit potential reduction abilities [180]. The most commonly used precursor for the green synthesis of gold nanoparticles is tetra-chloroaurate salt (HAuCl_4_), which provides gold ions (Au^3+^) subsequently reduced by the chosen reducing agents present in the synthesis method [180]. Some reducing agents employed in the green synthesis of gold nanoparticles exhibit stabilizing properties, effectively mitigating nanoparticle aggregation and ensuring colloidal stability. However, in certain instances, additional stabilisation measures become necessary to optimise the properties and functionality of the synthesised nanoparticles. In these cases, additional stabilisers, including but not limited to starch and chitosan, are used [181]. Biologically synthesizing metallic nanoparticles involves several controlling factors affecting nucleation and stabilisation. These factors include pH, solvent concentrations, reaction time, and temperature [182]. They may influence the size and morphology of synthesised nanoparticles, indicated by a change in the colour of the solution [183]. Methods for the synthesis of nanoparticles are presented in Figure 2.

### 5.2. Biotemplates Used for the Green Synthesis of Gold Nanoparticles

Biological methods for the synthesis of nanoparticles include utilizing substances, or even entire organisms, derived from natural sources, such as plants, fungi, bacteria, and microalgae. Bio-templates offer a sustainable and environmentally friendly alternative to traditional chemical methods, with their unique biochemical compositions [186]. These methods often limit the bioavailability of produced nanoparticles and generate hazardous wastes that could accumulate [187,188].

The synthesis of gold nanoparticles with parts of plants or their extracts has been proven effective and environmentally friendly. Diverse plant metabolites, such as terpenoids, flavonoids, sugars, and proteins, can actively reduce metal ions, forming nanoparticles [185]. This reducing agent can also play a role in stabilizing the nanoparticles created, which may prevent aggregation, ensure long-term storage capacity, and control the size and shape of particles [189]. Elia et al. used aqueous leaf extracts of *Salvia officinalis*, *Lippia citriodora*, *Pelargonium graveolens*, and *Punica granatum grains* to synthesise AuNPs [190]. In their study, they assessed the stability and biocompatibility of the particles, confirming both. The ability of aqueous *Aloe vera* extracts to synthesise gold and silver nanoparticles was proved by Chandran et al. [191]. The AuNPs they obtained varied in size, ranging from 50 to 350 nm, with a predominance of triangular shapes. Increasing the extract concentration resulted in more occurrences of spherical particles, as evidenced in UV-Vis-NIR absorbance spectra. GS of AuNPs and AgNPs can also be achieved with water extract of *Capsicum chinense* leaves. In their study, Rosales et al. [192] proved that, after synthesis, the level of polyphenols, reducing sugars, and amino acids decreased, indicating the crucial role of these compounds in GS. Another example of green synthesis using aqueous plant extract comes from Ghosh et al., who prepared golden nanoparticles from extracts of *Gnidia glauca* flowers [193]. The nanoparticle shapes were spherical, hexagonal, triangular, and trapezoidal. A great chemo-catalytic potential against 4-nitrophenol characterised these nanoparticles. Nayan et al. achieved similar chemo-catalytic potential with nanoparticles using water extract from *Mangifera indica* flowers to synthesise gold nanoparticles [194]. In a study by Cardoso-Avila et al., the water extract of *Rosa canina* L. found its application in the green synthesis of silver and gold nanoparticles [195]. The nanoparticle diameter was measured with TEM, with 26 nm for AuNPs and 34 nm for AgNPs, respectively. These gold nanoparticles were also characterised by their ability to catalyze 4-nitrophenol to 4-aminophenol. However, silver nanoparticles were measured using their antimicrobial abilities. In another study, Moosavy et al. synthesised AuNPs and AgNPs using essential oils from *Mentha spicata*, which acted as both reducing and stabilizing agents [196]. The average sizes of the silver and gold nanoparticles were evaluated to be 24 nm and 19.61 nm, respectively, with most spherical particles. Cytotoxicity in the HEPG-2 cell line, antioxidant effects and antibacterial activity against *Escherichia coli*, *Listeria monocytogenes*, *Salmonella Typhimurium*, *Staphylococcus aureus*, and *Bacillus cereus* were also measured. Benedec et al. used aqueous extracts from ethanol taken from the aerial parts and leaves of *Origanum vulgare* [197]. The obtained particles exhibited a quasi-spherical shape with a mean diameter of approximately 40 nm. They demonstrated significant antioxidant activity and potential inhibitory effects against *Staphylococcus aureus* and *Candida albicans*. These biocompatible nanoparticles also exhibited a plasmonic effect. Rahman et al. used methanolic extracts of *Ricinus communis* L. to synthesise golden nanoparticles [198]. These AuNPs exhibited significant antibacterial characteristics against *Escherichia coli*, *Pseudomonas aerueginosa*, *Klebsiella pneumoniae*, *Bacillus cereus*, *Salmonella Typhi*, and Methicillin-resistant *Staphylococcus aureus.* Coffea arabica is also commonly employed in green synthesis. Bogireddy et al. compared gold nanoparticles synthesised by the classical Turkevich method with those created by the green method, with *C. arabica* seeds aqueous extract [199,200]. In this study, the influence of pH during synthesis was also investigated. Additionally, the catalytic ability of the synthesised nanoparticles was assessed. In another study, Yust et al. compared the ability of spent coffee extracts prepared at different roast levels (ranging from medium to dark) and different brewing methods (hot brewing, cold brewing, and espresso brew) to synthesise gold and silver nanoparticles [201]. The nanoparticles ranged from 10 nm to 500 nm in diameter and exhibited various shapes.

The potential use of microorganisms in biological methods to synthesize nanoparticles is widely considered, due to their capacity to generate extracellular and intracellular materials [202]. The mechanism of biosynthesis can vary among different microorganisms. It often involves secondary metabolites, such as reducing agents or enzymes, facilitating reduction reactions [203]. In their study, Lim et al. used *Bacillus subtilis* cell-free extract to synthesize AuNPs [204]. In addition, they conducted a proteomic analysis to identify the protein responsible for the biogenic synthesis of AuNPs. They proposed a mechanism in which gold ions form bonds with sulfur-containing amino acids of catalase A, followed by the stabilisation of nanoparticles through peptide bonds formed during the thermal denaturation of the enzyme. In another study, Correa-Llantén et al. used *Geobacillus* sp. strain ID17 isolated from environmental samples from Antarctica to produce AuNPs [205]. Obtained nanoparticles accumulated intracellularly were primarily exhibiting quasi-hexagonal shapes and ranged in size from 5 to 50 nm. The metal-reducing ability of *Shewanella oneidensis* was analyzed in a study conducted by Suresh et al. [206]. This bacterium produced extracellular spherical AuNPs ranging from approximately 2 to 50 nm. These nanoparticles were evaluated for their antibacterial activity against *E. coli*, *S. oneidensis* and *B. subtilis* strains. However, the study did not demonstrate these activities.

Fungi have also been shown to be potential microorganisms in the biosynthesis of nanoparticles. In a study by Clarance et al., endophytic *Fusarium solani* fungi were isolated from the roots of the fragrant *Chonemorpha* plant. Later, the aqueous filtrate of *F. solani* was used for the green synthesis of golden nanoparticles with a 40–45 nm diameter. Those stable particles showed potential anti-cancer activity against MCF-7 and HeLa cell lines [207]. Zhang et al. synthesized gold nanoparticles with three fungus species: *Aureobasidium pullulans*, *Fusarium* sp. and *Fusarium oxysporum.* Their study indicated that reducing sugars led to the spherical morphology of intracellular AuNPs when proteins inflicted accumulation [208]. In another study, Das et al. produced gold nanoparticles with *Rhizopus oryzae* mycelia, with an average diameter of 10 nm. Those nanoparticles showed good adsorption of pesticides, and their antimicrobial activity against *P. aeruginosa*, *E. coli*, *B. subtilis*, *S. aureus*, *Salmonella* sp., *S. cerevisiae*, and *C. albicans* was assessed [63].

Regarding biomanufacturing nanoparticles using algae, their potential has been tested for many species, including blue-green algae, brown algae, red algae, green algae, and diatoms [209]. The ability of algae to accumulate heavy metals and other potentially toxic substances and eliminate them is well known [210]. Ramakrishna et al. used an aqueous extract of *Turbinaria conoides* and *Sargassum tenerrimum* brown algae to synthesize nanoparticles; their diameters ranged from 5 to 57 nm [211]. These nanoparticles were tested for their catalytic properties in reducing nitroarenes to amino-arenes. The aqueous extract of other brown algae, *Sargassum muticum*, has been used for the GS of stable gold nanoparticles by Namvar et al. In contrast, the average diameter of the obtained nanoparticles ranged from 3 to 8 nm [212]. Another study using the brown algae *Cystoseira baccata* as a potential source of AuNP synthesizing factor was conducted by González-Ballesteros et al. The obtained nanoparticles were characterized as stable, spherical, and polycrystalline, with an average size of approximately 8.4 nm. Those nanoparticles showed potential anti-cancer activities against human colon cancer cell lines: Caco-2 and HT-29 did not show a cytotoxic effect against the healthy fibroblastic cell line PCS-201-010 [213]. AuNPs were successfully synthesized using green microalgae *Chlorella vulgaris* extract by Annamalai and Nallamuthu. The size of these AuNPs ranged from 2 to 10 nm [214]. In addition, the potential antimicrobial activity of synthesized nanoparticles against *Candida albicans* and *Staphylococcus aureus* was investigated.

Mammalian cell lines are able to synthesize gold nanoparticles in a process called biomineralization [215]. Cellular gold nanoparticle biomineralization using gold ions could improve delivery within dense biological tissues, increase intracellular gold uptake, and enhance specificity for cancer cells, making it a viable strategy for clinical translation. Intracellular biomineralization using polyethylene glycol as a delivery vector for ionic gold can produce plasmonic gold nanoparticles in human breast cancer cell cultures (MCF-7) at micromolar concentrations within 30 min, as shown in a study conducted by Schwartz-Duval et al. [216]. Vectorized biomineralization of ionic gold can potentially enhance biomedical applications, further tested in given study within MCF7 tumor mouse xenografts.

### 5.3. Applications of Green Synthesised Gold Nanoparticles

The versatile applications of green synthesised gold nanoparticles, which vary depending on their shape, span multiple industries, including medicine, technology, chemistry, and industry [24]. The potential antimicrobial activity of gold nanoparticles against numerous microbial species is widely recognised [217]. Gold nanoparticles possess antimicrobial abilities due to oxidative stress induction, metal ion release, and non-oxidative processes, making them great alternatives to antibiotic-resistant bacteria treatment [218]. Another potential biomedical application is the anti-cancer activity of given nanoparticles. Green-synthesised AuNPs have anti-cancer abilities due to their prolonged circulation time, easy modification of ligands, and increased uptake through receptor-mediated endocytosis [219]. Other mechanisms of their anti-cancer ability include generating reactive oxygen species (ROS), leading to cell death-induced pathways [220]. The unique properties of gold nanoparticles, such as their high surface area-to-volume ratio, excellent biocompatibility, and surface plasmon resonance, make them ideal candidates for imaging applications [221] and biosensing [222,223,224]. For example, they can be used in cancer screening to detect the characteristics of CA 15-3 antigens of breast cancer [225] or potentially toxic substances, such as aflatoxin B1 [226]. Gold nanoparticles also show potential usage as nanocarriers for various molecules, such as in drug delivery systems [227]. In this process, molecules adhere to the surface of the gold particles and the entire complex is introduced into the cells. This introduction into cells can occur forcibly, as seen with gene guns, or naturally through particle ingestion. Within cells, molecules will eventually separate from gold particles [228]. As research in this area progresses, the potential for further innovation and development of eco-friendly nanomaterials remains high, paving the way for sustainable solutions to pressing environmental and health challenges. Continued research and development in this field is crucial to harness its full potential and address complex challenges in diverse industries.

## 6. Conclusions

Our critical review of the toxicity of AuNPs, focussing on toxicological aspects, safety evaluation, and green toxicology, reveals a nuanced landscape that underscores both the promising applications and potential risks associated with AuNPs. Through a systematic exploration of current research, we have identified key insights that highlight the complex interaction between the physico-chemical properties of AuNPs and their biological and environmental interactions.

The toxicological profile of AuNPs is intricately linked to their size, shape, surface charge, and coating, and studies indicate a spectrum of cellular responses, from minimal to significant toxicity. These responses are mediated by mechanisms such as oxidative stress and inflammation, although the pathways involved are often complex. Despite some studies suggesting a relatively benign profile for well-characterised AuNPs, the variability in regulatory standards and assessment methodologies across different applications, particularly in cosmetics, underscores the need for more standardised and comprehensive safety evaluations. The review emphasises the importance of conducting long-term and in vivo studies to better understand the implications of chronic exposure to AuNPs. In the emerging field of green toxicology, there is a clear directive for developing AuNPs that minimize environmental impact through sustainable synthesis methods and lifecycle assessments. However, our review identifies a significant gap in research focused on the long-term environmental fate, biodegradability, and potential ecotoxicity of AuNPs. Advancing green chemistry principles in developing and applying AuNPs is paramount to achieving safer and more sustainable nanotechnologies. The multifaceted nature of AuNP research requires a multidisciplinary approach that integrates toxicology, materials science, environmental studies, and regulatory policy. Future research should establish standardised protocols for synthesizing, characterizing, and testing AuNPs, conduct comprehensive long-term in vivo studies, and expand the scope of green toxicology research.

In conclusion, the field of AuNPs is marked by a dynamic potential and significant challenges. The promise of AuNPs across various domains must be balanced with a thorough consideration of their potential risks. Our review advocates for continued research, responsible innovation, and the development of regulatory frameworks that ensure the safety and sustainability of AuNPs. As we continue to exploit the benefits of AuNPs, it is critical to proceed with caution, guided by a commitment to precaution and responsible management in the face of uncertainty.

## Figures and Tables

**Table 1:** In vitro toxicity studies on AuNPs.

Organism	Effects	Particle	References
3T3 cells	Produce more reactive oxygen species than plain AuNPs	Monodispersed AuNPs of diameter 15 ± 1 nm	[33]
A549 and Vero cells	No toxicity	Citrate- and MUA-Coated Nanospheres of 13 and 60 nm and MUA-Coated Gold Nanostars of 60 nm	[34]
A549 cells	Intrinsic and extrinsic apoptotic pathways reflected in cell damage	AuNPsof diameter approximately 17 nm, coated with serum proteins	[35]
A549 cells	Cytotoxicity by substantial changes in nuclear morphology and nuclear condensation. Assumed circular shape because of the induced stress	AuNPs with an average dynamic diameter of 33 nm	[36]
A549 cells	An inflammatory response	BioPure^TM^ silver and gold nanoparticles with a diameter between 20 and 60 nm in a concentration of 1 mg/mL	[37]
AGS, A549, NIH3T3, PK-15, and Vero cells	Suppression of growth of cells in a dose-dependent manner by delay of cell cycle and induction of apoptosis	AuNPs of three sizes: (10 nm × 39 nm, 10 nm × 41 nm, 10 nm × 45 nm)	[38]
Balb/3T3 cells	Oxidative stress reflected in DNA damage but with reduced cytotoxicity	Spherical AuNPs of 12 nm diameter, uncoated and coated with hyaluronic acid in a concentration of 10 mg/mL in PBS	[39]
Balb/3T3 cells	Cytotoxicity by disruption of actin cytoskeleton	Citrate-stabilized AuNPs of 5 and 15 nm diameter in concentrations of 2, 10, 20, 39.2, 58.8 g/mL	[40]
C17.2 and PC12 cells	Induced oxidative stress by cell viability and deformations of actin and tubulin	4 nm diameter AuNPs in concentrations ranging from 10 to 200 nM.	[41]
Caco-2 cells	Did not produce acute cytotoxicity	AuNPsin concentration 0, 5, 10, 20, 40, 80, 125, 250, 500 or 1000 g/mL	[42]
CHO, BEAS-2B, and HEK293 cells	Exert higher toxicity	Citrate-stabilized AuNPs of diameter 14 nm (concentration 2.25 × 10^12^ nps/mL) and 20 nm (concentration 7.76 × 10^11^ nps/mL)	[43]
Epithelial cells of airways	Elevation of lipid peroxidase, DNA damage, and cytotoxicity	AuNPs of 20 nm diameter in concentration 1 nM/L	[44]
Granulose cells of the ovary	Induced an elevation in estrogen accumulation	10 nm AuNPs in concentration 2.85 × 10 ^10^/mL	[45]
HaCaT (Human keratinocyte cell line)	Cell death by apoptosis and necrosis	The average particle sizes are reported as follows: 1.8 ± 0.7 nm for neutral particles (MEEE), 1.6 ± 0.8 nm for positive particles (TMAT), and 1.8 ± 0.7 nm for negative particles (MES).	[46]
HEK293 cells	Modified gene expression and had no toxicity	Phosphine-stabilized and thiol-stabilized AuNPs of 1.4 nm diameter	[47]
HeLa and U937 cells	Cytotoxic	15, 40 and 80 nm Citrate-capped AuNPs in various concentrations	[48,49]
HeLa cells	No indication of cytotoxicity	AuNPs of diameter ranging from 4.0 to 5.4 nm in different concentrations	[50]
HeLa cells	No toxicity effects	Silica-coated AuNRs of diameter ranging from 4 to 16 nm in concentration 1–400 µg/mL and glucose-capped AuNPs of diameter within 5–9 nm at concentration 5.5 µM/mL	[51,52]
HepG2 and PBMC cells	In vitro cytotoxicity and genotoxicity effects at low concentrations	AuNPs capped with either sodium citrate (average diameter of 18.2 ± 0.4 nm) or polyamidoamine dendrimers (average diameter of 10.9 ± 0.4 nm) Concentrations from 0.01 to 50.0 M	[53]
HepG2 cells	AuNPs do not change the concentration of inflammatory markers compared to the control. Indicated tails moment similar to those from the positive control exposed to hydrogen peroxide	Citrate-stabilFd AuNPs with 10, 30 or 60 nm of diameter size. The concentration of 10 ppb and 10 ppm	[54]
HL-60 and HepG2 cell lines	Cytotoxic effects associated with reduction in GSH and increase in ROS	AuNPs with diameters of 30, 50 and 90 nm in concentrations 1–25 mg/mL	[55]
HL7702 cells (Human liver cell lines)	Early decrease in cytosolic GSH, depolarisation of mitochondrial transmembrane potential, and apoptosis	AuNPs with a diameter of 8 nm and 37 nm	[56]
HT29 cells (Human colorectal adenocarcinoma)	Significant reduction in viability of cells. However, no genotoxic effects	AuNPs with a diameter of 31.99 ± 0.16 nm and a concentration of 9.8 µg/mL	[57]
Human cell lines	Little or no immunotoxic, cytotoxic, and genotoxic effects	4.5 nm AuNPs in the concentration of 6.05 × 10^13^ nanoparticles/mL a	[58]
Human spermatozoa	Affects viability and motility	50 nm sized AuNPswith concentrations 30, 60, 125, 250 and 500 µM	[59]
L5178Y cells	No damage to the DNA at 60 nm but damage at 100 nm	4, 50, 100 and 200 nm sized AuNPs in concentrations of 0, 6.25, 12.5, 25, 50, 100 and 200 μg/mL	[60]
MDA-MB-231 cells (Breast cells)	Reduction in proliferation	1.9 nm spherical AuNPs (Aurovist™)	[61]
MG63 cells	Low long-term toxicity	AuNPs of diameter 10 nm in concentrations of 1 and 10 ppm	[62]
MRC-5 cells	Slight hepatotoxic and nephrotoxic	AuNPs capped with GNPC and GNPBwith an average diameter size of 15–20 nm and concentrations 51, 128, 320, 800, 2000 and 5000 ppm	[63]
MRC-5 cells	High lipid peroxidation, upregulation of antioxidants, expressions of protein and gene of stress response	20 nm diameter AuNPs in 1 nM concentration	[64]
Vero, MRC-5, and NIH/3T3 cells	Reduction in growth related to apoptosis and autophagy	Nano-rod structure with an average length of 10–40 nm with concentrations 0, 36, 72, 180, 360 and 720 ng/mL	[65]
Rat liver	Yield a great lipid peroxidation	AuNPs of diameter 10 nm. Doses of 50 µL of NP solution	[66]
Tumor ascites and normal peritoneal cells	No morphological changes and cell death	Functionalized AuNPs of diameter 4.5, 10 and 20 nm in concentrations 10, 25, 50 and 100 mM	[67]
Vero cells	No toxicological effects	Porphyran-reduced AuNPs with an average particle size of 14 ± 2 nm in concentrations 10, 50 and 100 µM	[68]

**Table 2:** In vivo toxicity studies with AuNPs.

Organism	Effects	Particle	References
BALB/c mice	Apoptosis and inflammation of liver tissue	13 nm PEG-Coated AuNPs with the average injected numbers of particles per mice: 1.76 × 10^11^, 8.8 × 10^11^, and 4.4 × 10^12^ for low, middle, and high doses, respectively.	[95,96]
Broiler chicken	Caused recognisable oxidative damage to blood, histopathological changes, up-regulation of IL-6, expression of Nrf2 gene, fragmentation of DNA, a significant decrease in antibody titer against avian influenza (AI) and Newcastle disease (ND)	Gold nanoparticles colloidal solution (25 ± 5 nm)	[97]
*D. magna*	LC_50_ was reported as 2 mg/l after 48 h	Nanoparticles with a diameter of approximately 15 nm	[70]
*D. magna*, *T. arcticus*	LC_50_ was reported as 0.64 mg/l after 48 h for *D. magna* and 14.4 mg/l after 96 h for *T. arcticus*	0–10 mg/L concentration of Au^3+^	[98]
*Drosophila melanogaster*	Caused transmissible mutagenic effects	Citrate-capped 15 nm AuNPs in the concentration of 100 pM	[99]
*Drosophila melanogaster*	Sharp decline in fertility and life span, presence of DNA fragments, and strong over-expression of stress proteins	Citrate-capped 15 nm AuNPs in six different concentrations (1.9, 3.8, 19, 38, 190, and 380 pmol/L) dispersed in food	[100]
Female and male mice	Liver and kidney damage whose effects were sex-dependent. Damage to the neuronal system	Different diameters of AuNPs ranging from 3 to 100 nm	[101]
Female mice	Spherical AuNPs in live and macrophages	AuNPs of diameter 2, 4 and 100 nm in, respectively—15 × 10^13^ particles/mL, 9 × 10^10^/mL and the 100 nm 6 × 10^9^/mL.	[102]
Fetal mouse organs	No indication of toxicity in the fetus and placenta	20 and 50 nm AuNPs	[103]
Male CD1 mice	Accumulation at various parts of the brain	Protein and polyelectroylte coated AuNPs with a diameter of 15 ± 1 nm injected in the concentration of 144.5 nM	[104]
Male Wistar rats	AuNPs persist and accumulate in the spleen and liver	AuNPs of 20 nm diameter were injected at 15.1 µg/mL.	[11]
Male WU Wistar rats	Large particles of spherical AuNPs were observed in blood, spleen, and liver, while smaller particles were seen in the spleen, blood, thymus, lungs, liver, kidney, testis, heart, and brain	Gold nanoparticles have a 10, 50, 100 and 250 nm diameter. Injection concentration was respectively 77, 96, 89 and 108 µg/mL	[105]
ICR Mice	Lungs, kidney hemorrhage, lymphocytic infiltration, and inflammatory response	PEGylated 13 nm gold colloids	[87]
Mice	Liver damage	5, 10, 30, and 60 nm PEG-coated AuNPS dosed 4000 µg/kg	[106]
Mice	Apoptosis and acute inflammation	13 nm PEG-coated AuNPs. The mean quantities of particles injected per mouse were 1.76 × 10^11^, 8.8 × 10^11^, and 4.4 × 10^12^ for the low, medium, and high doses, respectively.	[96]
Mice	Affects kidney function and produces toxicity	GSH- and BSA-coated AuNCs with an average size of 2.1 nm. The injected concentration is up to 7550 µg/mL	[107]
Mice	Greatest toxicity and affecting organ index. Induced reduction in RBC, spleen index, and body weight	Citrate-capped AuNPs of diameter 13.5 nm in different concentrations varying from 137.5 to 2200 µg/kg	[93]
Mice	Produced no effect on normal growth	AuNPs capped with BSA and HSePEGeCOOH in a diameter of about 4 nm in various concentrations	[108]
BALB/C Mice	Caused loss of weight and appetite. However, smaller AuNPs did not produce any sickness	Naked colloidal AuNPs ranging in diameter from 3 to 100 nm injected intraperitoneally at a dose of 8 mg/kg/week	[5]
Mice (ddy)	AuNPs of all sizes were noticed in the spleen, liver, and lungs	AuNPs ranging in size from 15 to 200 nm administered in 1 g/kg intravenously	[109]
Pregnant C57BL/6 mice	Non-crossing of maternal-fetal barrier	2 and 40 nm AuNPs injected intravenously and 40 nm intraperitoneally	[110]
Rats	Accumulation in the spleen and liver	PEG-coated AuNPsofdiameter ranging from 11 to 31 nm injected in various concentrations	[111]
Rats	ROS-induced cytotoxicity that is size-dependent	PEG-coated AuNPs in diameter ranging in size between 6.2 and 61.2 nm	[112]
Rats	Distribution of AuNPs was observed in the testis, liver, and kidney. However, there were no effects on the testis, whereas mild changes were noticed in the kidney and liver sections	AuNPs with an average size of 50 nm and various concentrations	[113]
Wistar rats	Traces of AuNPs in the kidney, spleen, liver, intestine, urine, and feces. Smaller NPs induced greater effects on DNA damage	AuNPs of 10, 30 or 60 nm diameter injected 0.4 mL/day	[54]
Wistar rats	Accumulate in neurons, liver, spleen, kidney, and cross the blood-brain barrier; no toxicity	12.5 nm citrate-coated AuNPs in different doses—40, 200 and 400 µg/kg/day	[114]
Zebrafish embryo	Delay in the development of eyes and pigmentation	1.3 nm AuNPs (functionalized with TMATeAuNPs)in concentrations ranging from 0.08 to 50 mg/L	[7]

**Table 3:** Summary of cell line toxicity.

System	Cell Line/Model	Nanoparticle Characterization	Effect	Reference
Nervous System	Porcine brain microvascular endothelial cells (pBMECs)	Diameter of 3 nm and 5 nm in concentration 15 µg/mL	No significant secretion of pro-inflammatory mediators (IL-1b, TNFα, PGE2) or negative effect on BBB integrity	[139]
Nervous System	human embryonic stem cells (hESCs) and neural derivatives	Diameter of 1.5 nm and 4 nm in six different concentrations ranging from 0.001 to 10 µg/mL	Pronounced neurotoxic effects, significant cell death, disruption of neural differentiation, altered DNA methylation patterns	[140]
Digestive System	Caco-2 cells (model for human intestinal epithelium)	Diameter less than 100 nm. Various concentrations of 0, 5, 10, 20, 40, 80, 125, 250, 500 or 1000 µg/mL	Internalisation without significant cytotoxic effects or oxidative stress	[42]
Digestive System	Model intestinal epithelial cell line	A diameter of 15 nm AuNPs dosed in the concentration of 50 ppm	Size-dependent absorption, accumulation, and cytotoxic effects, including mitochondrial dysfunction	[144]
Respiratory System	Human lung fibroblast cell line MRC-5	Diameter of 1 nm. Cells treated with 1 nM concentration.	Induced oxidative stress, formation of autophagosomes, increased expression of autophagy-related proteins	[64]
Cardiovascular System	Human umbilical vein endothelial cells (HUVEC)	Diameter of approximately 20 nm Cells treated with about 1 × 10^11^ AuNPs.	No significant cytotoxicity, no lysis of human erythrocytes or apoptosis/necrosis of endothelial cells	[148]
Urinary System	Human kidney cells (HK-2)	Diameter of 13 nm and 60 nm. The concentrations ranging from 1 μM to 60 μM	13 nm nanospheres (particularly coated with MUA) exhibit the highest toxicity, affecting mitochondrial function and inducing programmed cell death	[149]
Urinary System	HK-2 and 786-0 cells	Diameter of 5 nm and 200 nm. Cells treated in the concentrations of 1 and 10 μg/mL	Induced apoptosis and inhibited cell proliferation, differential effects on cellular parameters and signalling pathways	[150]
Sensory Organs	Keratinocyte cell line (HaCaT) and human epidermoid skin cancer cell line (A431)	AuNPs were synthesised with *Vitis vinifera* seed extract with a diameter ranging from 40 nm to 55 nm. Cells treated with 5, 10, 15, 20, 25 µM concentration	No cytotoxicity against HaCaT cells, cytotoxicity against cancer cells with increased reactive oxygen species and induction of apoptosis	[151]
Reproductive System	Human sperm	Diameter of 9 nm. Concentration of 44 ppm.	25% of sperm lost mobility, evidence of nanoparticle penetration into sperm heads and tails	[152]

## Data Availability

The data, analytical methods, and study materials that support the findings of this study are available from Kamil Jurowski (oksykologia@ur.edu.pl) on reasonable request.

## Abbreviations

AuNPs	gold nanoparticles
GS	green synthesis
ROS	reactive oxygen species
GSH	glutathione
HESCs	human embryonic stem cells
CNS	central nervous system
PLT	platelet count
MPV	mean platelet volume
PCT	plateletcrit value,
PDW	platelet distribution width
PEG	polyethylene glycol
WBC	white blood cells
RBC	red blood cells
